# *Streptococcus* strain D19^T^ as a probiotic candidate to modulate oral health

**DOI:** 10.1186/s12866-023-03066-7

**Published:** 2023-11-16

**Authors:** Wen Xiao Zhang, Chun Ling Xiao

**Affiliations:** 1 Microbiology Department of the First Affiliated Hospital of Hebei North University, Zhangjiakou, Hebei People’s Republic of China; 2 Key Lab of Environmental Pollution and Microecology of Liaoning Province, Shenyang Medical College, No. 146, Huang he North Street, Shenyang, Liao Ning People’s Republic of China

**Keywords:** *Streptococcus*, Broad spectrum antagonistic, *Staphylococcus aureus*, Adhesion inhibition

## Abstract

**Background:**

As probiotics protect host cells, they are used to treat bacterial infections. It has been indicated that probiotics may prevent or reduce the attachment of pathogens to host cells. In this study, *Streptococcus* strain D19^T^ was isolated from the oropharynx of a healthy child, and its adhesion performance and *Staphylococcus aureus* adhesion inhibition effect were analysed using human bronchial epithelial (16-HBE) cells, as an in vitro cell model. We evaluated the probiotic properties of the D19^T^ strain based on its acid–base, bile salt, and lysozyme tolerance; antibacterial activity; cytotoxicity; antibiotic sensitivity; in vitro adhesion to 16-HBE cells; and competitive, exclusion, and displacement effects against *S. aureus*.

**Results:**

*Streptococcus* strain D19^T^ showed tolerance to a PH range of 2–5 and 0.5–1% bile. However, it was more tolerant to 0.5% bile than to 1% bile. The strain also demonstrated an ability to adapt to maladaptive oropharyngeal conditions (i.e., tolerating 200 µg/mL lysozyme). It was resistant to 0.8 mM H_2_O_2_. The results also demonstrated that D19^T^ exhibited inhibitory activities against various common pathogenic bacteria. Furthermore, D19^T^ was not toxic to 16-HBE cells at different multiplicities of infection and was sensitive to most antibiotics tested. The adhesion rate of D19^T^ cells to 16-HBE cells was 47% ± 1.2%, which was significantly higher than that of *S. aureus* to 16-HBE cells. The competition, exclusion, and displacement assay results showed that D19^T^ has good inhibitory effect against *S. aureus* adhesion.

**Conclusions:**

The present study revealed that *Streptococcus* strain D19^T^ has the potential to be developed as a respiratory microbiota preparations.

## Background

Microbial communities exist on all surfaces of the human body, including the respiratory mucosa. Specialized bacterial communities inhabit particular sites of the respiratory tract and play important roles in maintaining human health [1]. Beneficial bacteria strongly affect the metabolism, nutrition, physiology, and immune functions of their hosts [2]. Improper use of antibiotics can lead to the development of drug-resistant strains [3].

Hundreds of microorganisms inhabit the human oral cavity [4].

Most of these microorganisms are commensals, while others are mutual symbionts with oral mucosal barrier functions. They confer resistance to pathogenic bacterial colonization in the host [5]. The Oropharyngeal microbiota dynamic and diverse. Various risk factors, such as poor dietary habits and poor oral hygiene, can alter the oral microbiota and disrupt the balance between symbiotic and pathogenic microorganisms [4]. These distortions may lead to opportunistic pathogens dominating the oral cavity, leading to pharyngitis, dental caries, gingivitis, and other oral diseases and infections [6]. The use of bacteria from the pharynx of healthy individuals as probiotics is considered safe [7]. Probiotics are living microorganisms that, when applied at appropriate amounts, can be beneficial to host health [8]. Probiotics can maintain the balance of the microbiota and inhibit the growth of pathogenic bacteria [9]. The respiratory tract, as a lumen that communicates with the outer environment, has dominant taxa in its microbiota, such as the partially isolated type A haemolytic *Streptococcus* spp. (*S. salivarius* and *S. oralis*) in the human oropharynx. These *Streptococcus* spp. are the dominant components of the microbiota in the upper respiratory tract and have a high affinity for human mucosa, protecting epithelial cells from pathogen adhesion [10]. The ability of probiotics to adhere to host cells is a classic selection criterion. Such probiotics can compete against pathogens for host cell binding sites and inhibit pathogenic bacterial adhesion [11]. In addition, the excellent adhesion ability of probiotics enables them to interact with the host and have beneficial effects. *Staphylococcus aureus* is a pathogenic bacterium in the mouth. It usually leads to microecological disorders and oropharyngeal dysfunction [12]. Therefore, the oropharyngeal tract is a potential target for developing new probiotic products. The aim of this study was to investigate *Streptococcus oropharyngis* strain D19^T^ as a candidate probiotic and analyse its potential probiotic characteristics in vitro for further regulate oral health.

## Results

### Strain identification

The 16 S rRNA gene sequence of the novel strain was obtained by sequencing (1433 bp), uploaded to the GenBank database (MN061029) and compared with the sequence in GenBank by the NCBI server using BLAST. As a result, compared with strain D19^T^, the type strains of Streptococcus with 16 S rRNA similarities greater than 97% were subsp. The results showed that *Streptococcus oralis* subsp. dentisani DSM 27088T, Strep-tococcus mitis ATCC49456T, *Streptococcus pneumoniae* ATCC 33,400 T, *Streptococcus pseudopneumoniae* ATCC BAA-960 T, and *Streptococcus oralis* ATCC 3503 T were most closely related to strain D19^T^.

### Resistance to acidic pH, bile, lysozyme, and H_2_O_2_

**Fig. 1:**
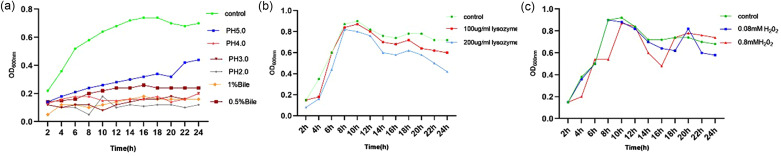
Growth curve plotting for *Streptococcus* strain D19T under different conditions. (**a**) Acidic pH and 0.5% or 1.0% bile. (**b**) 100 µg mL-1 or 200 µg/ mL-1 lysozyme. (**c**) 0.08 mM or 0.8 mM hydrogen peroxide (H2O2). OD = optical density

We studied the potential probiotic properties of *Streptococcus* strain D19^T^. A probiotic must be resistant to oropharyngeal stress conditions to maintain its activity and viability in this site. Strain survivability at various pH levels and bile salt concentrations is shown in Fig. 1(a). D19^T^ could tolerate a PH range of 2–5. D19^T^ had greater resistance to 0.5% bile than to 1% bile. Moreover, it tolerated 0.08, and 0.8 mM H_2_O_2_, and 100 and 200 µg/mL lysozyme. Hence, D19^T^ could overcome the hostile conditions of the oropharyngeal tract (Fig. 1(b) and 1(c)). It should be able to tolerate oral and digestive tract conditions, adhere to epithelial membranes, and compete with other microbes [19].

### Antimicrobial activity

Table 1 shows the antagonism of *Streptococcus* strain D19^T^ against eight common pathogenic bacteria. It was effective against *S. aureus*, *Pseudomonas aeruginosa*, *Escherichia coli*, *Streptococcus pneumoniae*, *Proteus vulgaris*, *Streptococcus pyogenes*, *Acinetobacter baumannii*, and *Klebsiella pneumoniae*.

**Table 1:** Antimicrobial activity of *Streptococcus* strain D19^T^

Antagonistic strain				Pathogen				
	*S.aureus*	*P.aeruginosa*	*E.coli*	*K.pneumoniae*	*P.vulgaris*	*E.cloacae*	*A.baumannii*	*S.pyogenes*
D19^T^	+++	+++	++	++	++	++	++	++

+++: bacteriostatic zone diameter ≥ 20 mm; ++: 15 mm ≤ inhibition zone diameter < 20 mm.

### Antibiotic susceptibility testing

Table 2 shows the sensitivity of D19^T^ to eleven different antibiotics. D19^T^ was sensitive to penicillin, ampicillin, cefepime, cefotaxime, ceftriaxone, linezolid, clindamycin, chloramphenicol, minocycline, tetracycline and vancomycin. It was resistant to kanamycin and norfloxacin.

**Table 2:** Antibiotic susceptibility of *Streptococcus* strain D19^T^

Antibiotic	Dose (µg/mL)	Sensitivity
Penicillin	30	S
Ampicillin	10	S
Cefepime	30	S
Cefotaxime	30	S
Ceftriaxone	30	S
Linezolid	30	S
Clindamycin	2	S
Chloramphenicol	30	S
Vancomycin Kanamycin Norfloxacin Minocycline Tetracycline	30 30 10 30 30	S R R S S

S = susceptible; I = intermediate (moderately resistant); R = resistant

### Cytotoxicity assay

**Fig. 2:**
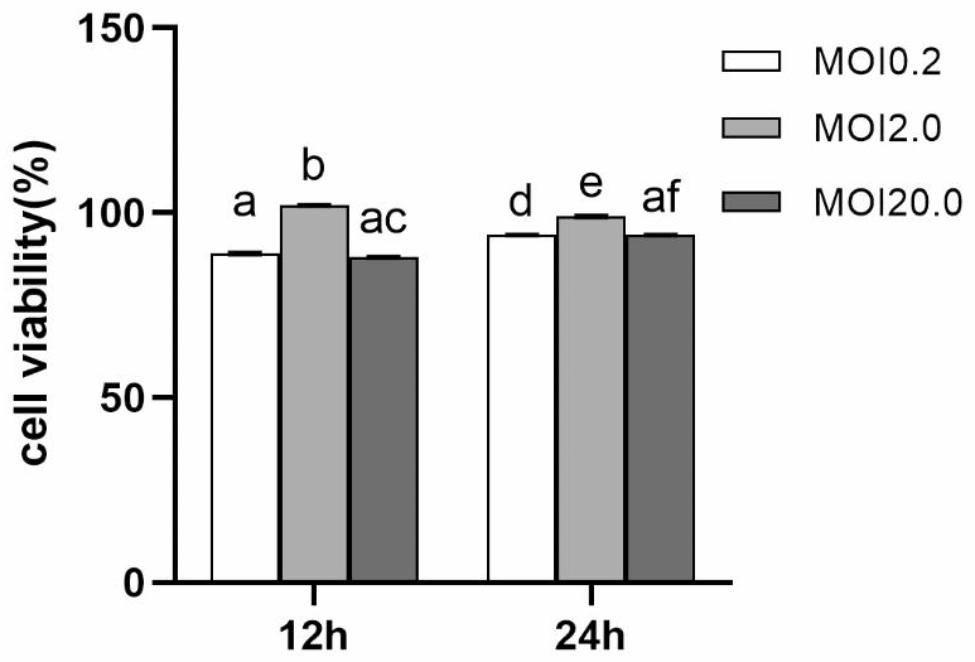
Effect of D19T on 16-HBE cell proliferation—at different multiplicities of infection (MOIs). The MOIs were = 0.2, 2, and 20. Means with different letters (a–f) differ significantly (P < 0.05)

A key determinant of the probiotic effects of strains is their ability to adhere to host epithelial cells. Therefore, we aimed to determine the toxic effects of the probiotic strain on epithelial cells. As shown in Fig. 2, there was no significant change in cell viability after coincubation of D19^T^ with human bronchial epithelial (16-HBE) cells at different multiplicities of infection (MOIs) for 12 h. However, after 24 h of incubation at an MOI of 0.2, D19^T^ showed > 100% cell viability. In addition, at an MOI 2.0 or 20.0, D19^T^ was not cytotoxic to 16-HBE cells. These findings are similar to those previously reported [13].

### Adhesion rate of strain D19^T^

**Fig. 3:**
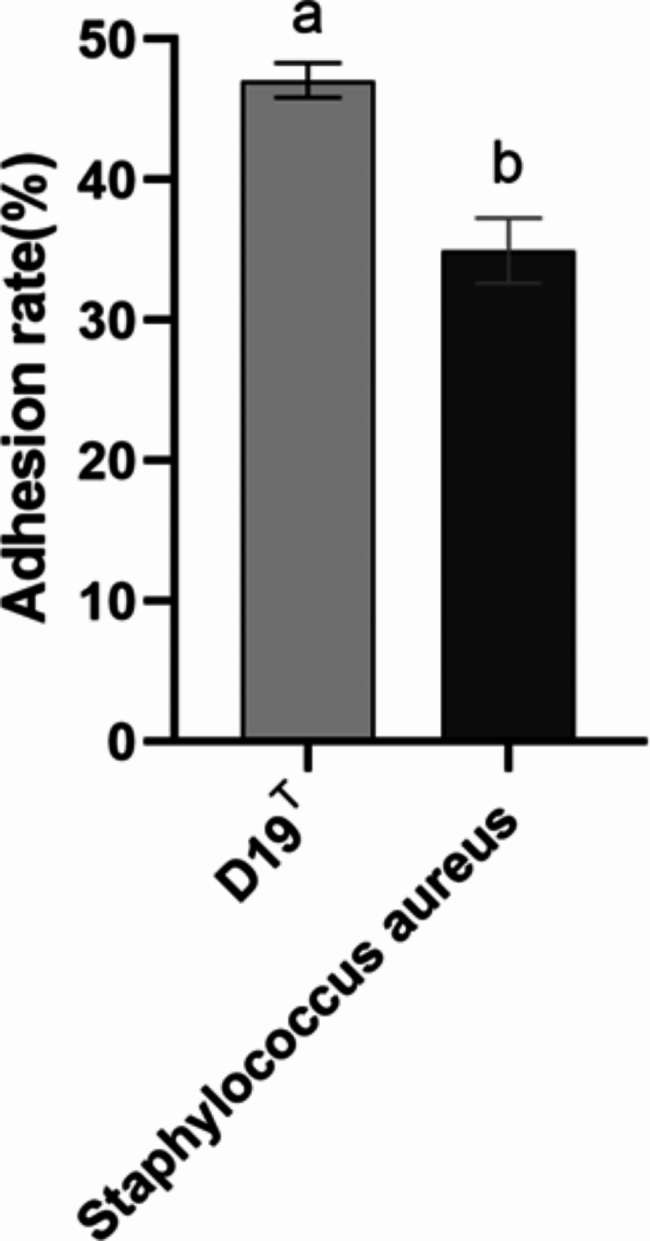
Rate of adhesion of strain D19T and *Staphylococcus aureus* to 16-HBE cells

The adhesion capacity of beneficial bacteria and pathogens may be affected by the in vitro cell line used for evaluation as well as the mechanisms underlying the interactions of the strain with the surface components of cells. As shown in Fig. 3, of the two strains tested, the adhesion rate of D19^T^ was higher than that of *S. aureus*, with D19^T^ showing an adhesion rate of over 40%.

### Effect of D19^T^ on *S. aureus* adhesion to 16-HBE cells

The adhesion inhibition effect of probiotics on pathogens is also an important indicator of strain quality. As shown in Table 3, *Streptococcus* D19^T^ prevented the adhesion of *S. aureus* through competitive action, with a relative competitive rate of 62.67%. Moreover, the adhesion rate of *S. aureus* to 16-HBE cells was significantly reduced (*P* < 0.01). Similarly, D19^T^ reduced the adhesion of *S. aureus* to 16-HBE cells through displacement, with a relative displacement rate of 52.24%. However, D19^T^ also reduced the adhesion of *S. aureus* to 16-HBE cells via exclusion. Studies have shown that probiotics compete with pathogens for adhesion sites because both probiotics and pathogens have similar types of adhesins on their surfaces. The inhibitory effect of D19^T^ on *S. aureus* adhesion in this study was relatively satisfactory. However, further research is needed on adhesion inhibition mechanisms to provide a theoretical basis for the development of respiratory microbiota preparations.

**Table 3:** Effect of D19^T^ on *Staphylococcus aureus* adhesion to 16-HBE cells

Strain		Competitive assay	Exclusion assay	Displacement assay
*Staphylococcus aureus*		47.28 ± 1.03	47.28 ± 1.03	47.28 ± 1.3
D19^T^		17.65 ± 1.35**	42.36 ± 1.74**	22.58 ± 1.02**

Note: Compared with *Staphylococcus aureus*, ** indicates a significant difference (P < 0.01) and * indicates a significant difference (P < 0.05)

## Discussion

In this study, a total of 1080 strains of bacteria were isolated from the oral and pharyngeal parts of children, among which strain D19^T^ had the best antibacterial effect. Choosing the most beneficial organisms in vitro, as an inexpensive and rapid detection approach, is more effective than choosing organisms in vivo. Although it is not possible to replicate all in situ conditions of the oropharyngeal ecosystem under in vitro conditions, in vitro detection is still a powerful approach for rapid screening of high potential strains, and extensive research can be conducted on many isolated strains to identify the specific characteristics of probiotic strains. Potential oral and pharyngeal probiotic strains are expected to tolerate oral and pharyngeal stress conditions, thereby improving the health of the host. The ability to resist acid, bile salt, H_2_O_2_, and lysozyme is considered a good indicator of the survival of oropharyngeal strains [13]. Antibacterial activity against pathogens is another important feature to be considered when selecting potential probiotic strains to maintain a healthy microbial balance in the body. This antagonistic activity is mainly attributed to the antibacterial substances or metabolites produced by probiotics, such as organic acids, bacteriocins, bacteriocin-like components, and H_2_O_2_ [14]. In this study, although the inhibition zones of the eight kinds of pathogenic bacteria were different, D19^T^ showed antibacterial activity against all of the strains. Among the bacteria, the antibacterial activity against *S. aureus* and *P. aeruginosa* was the strongest, with an inhibition zone of > 20 mm, which may be attributed to the production of H_2_O_2_ or bacteriocin by D19^T^. An ideal probiotic strain for human use must be derived from humans, lack potential virulence genes, and be sensitive to commonly used antibiotics [15]. In this study, strain D19^T^ was isolated from the oropharynx of a healthy child; it was identified as a *Streptococcus mitis* strain [16]. Owing to the difficulty in evaluating the adhesion performance of probiotics to host cells in vivo, many scholars worldwide use human-derived cell lines as in vitro models to study the adhesion ability of probiotics [17]. In recent years, multiple studies have reported that the microbiota in the lower respiratory tract is similar to that in the oropharynx; therefore, in this study, the human bronchial epithelial cell line 16-HBE was used as an in vitro cell model [18]. To understand the possible cytotoxic effects of strain D19^T^ on 16-HBE cells, we evaluated the viability of 16-HBE cells in the presence of D19^T^ at different MOIs (0.2, 2, and 20 ).We found that the D19^T^ strain had no toxic effect on 16-HBE cells at an MOI of 2.0 or 20.0, similar to previous study results, indicating that it is relatively safe [19]. The susceptibility of all potential probiotic strains to a range of commonly used human antibiotics should be evaluated to identify potential probiotic strains with transferable antibiotic-resistance genes that may be harmful to the host [20]. The tested strain D19^T^ was found to be sensitive to eleven commonly used antibiotics. Antibiotic-resistant bacteria have been generally considered unsafe for use as probiotics. Here, D19T was susceptible to most antibiotics, and the result was comparable to that of a previous study [21].The ability to adhere to host cells has been a classic selection criterion for potential probiotics; adherence may lead to brief colonisation, which helps promote immune regulation and stimulate the intestinal barrier and metabolic function. Jia et al. used fluorescence labelling to study the adhesion of the potential probiotic *Lactobacillus salivarius* AR809, isolated from the oropharynx, to FaDu cell monolayers. They found that it exhibited strong adhesion performance to FaDu cells. Additionally, one strain of *S. oropharynges* exhibited has a high adhesion ability, which was higher than previously reported values of 0.9% and 20% [22]. This higher result may be closely related to adhesins, secreted proteins related to adhesion, on the surface of the cells. The levels of these proteins are largely inconsistent, which leads to differences in the adhesion performance of strains. Adhesion and invasion of host tissues are essential steps in the pathogenesis of many pathogens and viruses [23]. It has been confirmed that probiotics can effectively inhibit cell binding and pathogen invasion [24]. In this study, strain D19^T^ inhibited the adhesion of *S. aureus* to 16-HBE cells through competition, exclusion, and replacement. These results indicate that strain D19^T^ may form a barrier through self-aggregation and adhesion mechanisms and may prevent potential pathogen binding to host cell receptors and coaggregation with potential pathogens, thereby protecting the host epithelium.

## Conclusion

We isolated strain D19^T^ from the oral cavity of a child and demonstrated its potential probiotic properties. Thus, it is a good probiotic candidate for improving oral health. Owing to its unique probiotic and functional properties, D19^T^ has the potential to protect the oropharyngeal tract against invading pathogenic microbes, thereby preventing pharyngeal infections. Moreover, D19^T^ has potential for use as a probiotic in health-promoting foods.

### Methods

#### Bacterial strains and culture conditions

Bacterial samples were collected from a throat swab. D19^T^ was obtained from the oropharyngeal mucosa of a healthy 6-year-old child. *Staphylococcus aureus* CGMCC10201, *P. aeruginosa* CGMCC10104, *E. coli* CGMCC10003, *K. pneumoniae* CGMCC31001, *P. vulgaris* CGMCC1.1651, *E. coli* cGMCC1.8726, *A. baumannii* CGMCC1.10395, and suppurative *Streptococcus* cGMCC9801 were purchased from the Shanghai Industrial Strain Preservation Center, China.

*Streptococcus* D19^T^ (BHI; HyClone Laboratories, Logan, UT, USA) was incubated in culture medium at 37 °C for 24 h, and then stored at -40 °C for later use. The pathogens used in the antagonism and adhesion inhibition experiments were cultured in BHI medium for 6–8 h.

### Isolation and identification of the strain

A throat swab sample was collected from the oropharynx of a healthy 6-year-old child from Shenyang, China, to isolate strain D19^T^. The sample was stored at -80°C until isolation and culture of D19^T^. The culture was placed in 1 mL of normal saline, mixed, and diluted by 10^− 3^ in a vortex mixer. Thereafter, 0.1 mL of the culture was evenly coated on BHI agar containing 5% defibrillated sheep blood. After incubation at 37°C for 24 h, white α haeemolytic colonies were extracted from the medium and purified using the continuous streaking technique. The phenotypic, phylogenetic, and genomic characteristics were analysed. The strain was identified using polymerase chain reaction (PCR). The total DNA was extracted and the primers used for 16S rRNA gene amplification were 27F/1492R (5’-agagtttgatcmtggctcag-3’ and 5’-ggytaccttgttacgactt-3’). PCR was performed using a 30 µL reaction mixture. The specific operation procedure was as follows: DNA degeneration (94 °C, 5 min), modification (94 °C, 30 s), annealing (56 °C, 30 s), extension (72 °C, 1 min 40 s), and final stretch (72 °C, 8–10 min). The PCR products were analysed using 1.5% agarose gel electrophoresis, and the sequencing of the amplicons was outsourced (Shanghai, China). The identification results showed that the isolate was a gram-positive, catalase-negative strain of a new *Streptococcus* species, closely associated with oral *S. pneumoniae* subspecies dentisani DSM27088, and was named *Streptococcus shenyangsis* sp. nov. [16].

### Resistance to acid and bile salts

The pH tolerance assay reported by Jia et al. (2019) was used with slight modifications. An overnight D19^T^ culture with a density of 10^8^ CFU/mL was inoculated in either BHI broth at 3% (v/v), with the pH adjusted to 2.0, 3.0, 4.0, and 5.0 with 1 M HCl or BHI broth containing 0.5% (w/v) and 1% (w/v) bile. Uninoculated BHI broth (pH 7.0) served as the control. The inoculated broths were incubated at 37 °C for 24 h. Every 30 min, the optical density value at 600 nm (OD600nm) was measured using an automatic growth curve analyser (Bioscreen C, Helsinki, Finland) after shaking the culture for 10 s.

### Resistance to lysozyme and H_2_O_2_

Overnight cultures of D19^T^ (1 × 10^8^ cfu/mL) were inoculated at 3% (vol/vol) into MRS broth containing lysozyme (100 and 200 µg/mL) or hydrogen peroxide (H_2_O_2_, 0.08 and 0.8 mM; Sangon Biotech), and incubated at 37 °C for 24 h. Inoculated MRS broth without lysozyme and H_2_O_2_ was used as a control. Every 30 min, the OD600nm value was measured using an automatic growth curve analyser after shaking for 10 s.

### Antimicrobial activity

Strain D19^T^ was cultured on a fibrillated sheep blood agar plate for 24 h, and monoclonal colonies were inoculated in BHI liquid medium and cultured on a 37 °C shaking table at 180 rpm for 24 h. The final concentration of the culture was adjusted to 1 × 10^9^ CFU/mL. Simultaneously, the indicator strains (*S. aureus*, *P. aeruginosa*, *E. coli*, *K. pneumoniae, K. vulgaris*, *E. coli*, *A. baumannii*, and *S. pyogenes*) were cultured to the logarithmic phase, and their concentrations were adjusted to 1 × 10^6^ CFU/mL. Finally, the inhibitory effect of strain D19^T^ on the indicator strains was observed using the Oxford cup method. Specifically, 100 µL of indicator bacterial solution was coated on a nutritional agar plate with sterile cotton swabs. Four Oxford cups were placed on the agar plate with a pair of sterile tweezers at equal distances. Thereafter, 200 µL of culture suspension of each antagonistic strain was added into two cups, and an equal amount of BHI medium of equal amount was added into the other two cups as controls. Finally, the culture plate was placed horizontally in a 37 °C incubator for 18 h. Three plates were used for each indicator strain.

### Antibiotic susceptibility

The antibiotic susceptibility of D19^T^ was determined using the disc diffusion assay according to the 2018 recommendations of the Clinical Laboratory Standards Institute [25]. Colonies were selected and cultured for 24 h on agar plates containing 5% (w/v) defibrinated sheep blood and suspended in 5 mL of sterile normal saline solution with a turbidity of 0.5 McFarland. A bacterial suspension was applied to MH medium with sterile cotton swabs. The antibiotics tested were penicillin (30 µg), ampicillin (10 µg), cefepime (30 µg), cefotaxime (30 µg), ceftriaxone (10 µg), linezolid (30 µg), clindamycin(2 µg),chloramphenicol(30 µg),kanamycin(30ug), ,norfioxacin(10ug),minocycline(30ug), tetracycline(30ug)and vancomycin (30 µg). All assays were performed in triplicate.

### Cytotoxicity assay

The cytotoxicity of D19^T^ in 16-HBE cells was determined using the colorimetric assay with 3-(4,5-dimethylthiazol-2-yl)-2,5-diphenyltetrazolium bromide (MTT) [13]. The cells were seeded in a 96-well tissue culture plate at a density of 8 × 10^4^ /well and grown until confluence. Thereafter, D19^T^ was added at MOIs (bacteria:16-HBE cells) of 0.2:1, 2:1, and 20:1 before coincubation at 37 °C under 5% CO_2_ for 24 h. Subsequently, 10 µL of MTT (5 mg/mL) was added to each well, and the suspensions were incubated at 37 °C under 5% CO_2_ for 4 h. After incubation, 150 µL of dimethyl sulfoxide was added to each well and the formazan crystal product was completely dissolved by shaking for 10 min. The absorbance (A) of the sample in each well was measured at 570 nm using a microplate reader (TECAN Infinite 200 PRO; Beijing Long yue Biological Technology Development Co., Ltd., CA, USA) at 570 nm. Cell viability was calculated as follows:

Cell viability (%) = (Asample/Acontrol) × 100 (1);

where, Asample is the absorbance of 16-HBE cells coincubated with D19^T^ and Acontrol is the absorbance of D19^T^ alone.

### Bacterial fluorescent labelling

The bacteria were labelled according to a previously reported method [26]. The bacterial strains were cultured to the middle and late periods of exponential growth and centrifuged at 4 °C for 10 min at 5000 *× g*. Thereafter, the supernatant was discarded; the pellet was washed twice with phosphate-buffered saline (PBS; pH 7.4), centrifuged at 4 °C for 10 min at 5000 *× g*, and mixed with carbonate buffer (0.5 mol/L, pH 9.5) to prepare 0.5 × 10^9^ CFU/mL bacterial suspensions. FITC solution was added at a final concentration of 50–100 µg/mL in the bacterial suspension; the solution was stirred at room temperature for 1–2 h and centrifuged at 5000 *× g* for 10 min. Subsequently, the supernatant was discarded, and the pellet was washed twice with PBS and centrifuged at 5000 *× g* for 10 min. The precipitated bacteria were suspended in PBS to obtain a bacterial suspension with a concentration of 1 × 10^9^ CFU/mL. The labelling was confirmed using fluorescence microscopy, and the bacterial suspension was stored at 4 °C, protected from light, until further use.

To observe the bacterial fluorescence signal, 5 µL of the labelled bacterial suspension was applied to a slide, and covered with a cover glass, and the bacterial fluorescence image was observed under a fluorescence microscope; a clear bacterial fluorescence field indicated successful labelling.

### Cell culture assays

The 16-HBE cell line was purchased from Shenyang Medical College (Liaoning, China). The cells were cultured in endothelial cell medium (ECM; HyClone) containing 5% (v/v) foetal bovine serum, 1% (w/v) penicillin/streptomycin solution, and 1% (w/v) endothelial cell growth supplements at 37 °C under 5% CO_2_. The cells were then cultured in a 25-cm^2^ flask containing 0.25% (v/v) trypsin-EDTA solution (Sigma-Aldrich Corp., St. Louis, MO, USA) at 37 °C for 13 min and centrifuged (4000 *× g*, 4 °C, 1 min). The cells were inoculated in a six-well tissue culture plate at a density of 8 × 10^4^/well and subcultured at 37 °C under 5% CO_2_ for 3 days until fusion.

### Adhesion experiment

The method described by Jia et al. [13] was used. Cell cultures without bacteria were used as blank controls. The experimental group contained 100 µL of culture medium and 100 µL of fluorescently labelled D19^T^ and *S. aureus* (1 × 10^9^ CFU/mL) in 96-well plates. The plates were placed in an incubator at 37 °C under 5% CO_2_ for 2 h, and then, washed with sterile PBS three times. The unattached bacterial cells were eluted, 0.1 mL of trypsin was added to each well, and the plates were incubated for 13 min. After the complete exfoliation of cells, 0.4 mL of complete culture medium was added to terminate the reaction. The liquid was collected, and its fluorescence intensity was measured using a microplate reader. The excitation wavelength was set at 495 nm, and the emission wavelength was set at 530 nm. Relative fluorescence intensity was determined using 10 replicates for each group.

Calculation formula:

Adhesion rate of bacteria (%) = A/A_0_ × 100;

where, A is the relative fluorescence intensity of the cell suspension after the adherence of D19^T^ and *S. aureus* cells to 16-HBE cells and elutes, and A_0_ is the relative fluorescence intensity of the cell suspension before the adherence of D19^T^ and *S. aureus* cells to 16-HBE cells.

### Competitive test

D19^T^ cells (unlabelled), 16-HBE cells, and pathogenic bacteria (FITC-labelled) were incubated at 37 °C under 5% CO_2_ for 2 h. Thereafter, the samples were washed with sterile PBS twice to remove nonadherent cells and allowed to stand in the dark.

### Exclusion test

D19^T^ cells (unlabelled) and 16-HBE cells were incubated at 37 °C under 5% CO_2_ for 1 h. Thereafter, the cells were washed with sterile PBS twice to remove nonadherent cells. Subsequently, pathogenic bacteria (FITC-labelled) were added, and the samples were incubated under 5% CO_2_ at 37 °C for 1 h.

### Displacement assay

Pathogenic bacteria (FITC labelled) and 16-HBE cells were coincubated at 37 °C under 5% CO_2_ for 1 h and washed with sterile PBS twice to remove nonadherent bacterial cells; thereafter, D19^T^ cells (unlabelled) were added, and the samples were incubated at 37 °C under 5% CO_2_ for 1 h.

After the above treatment, 0.1 mL of pancreatic enzyme was added to each culture well and the samples were incubated for 13 min. After complete shedding of cells, 0.4 mL of complete culture medium was added to terminate the reaction. The suspension was collected, and its fluorescence intensity was measured using an enzyme-linked immunosorbent assay. Each group had six replicates and each culture well had one replicate.

Calculation formula: Adhesion rate (%) = A_2_ / A_1_ × 100;

where, A_1_ represents the relative fluorescence intensity of *S. aureus* adhering to 16-HBE cells in the presence of strain D19^T^, and A_2_ represents the relative fluorescence intensity of *S. aureus* adhering to 16-HBE cells in the presence of strain D19^T^.

### Statistical analysis

All experiments were conducted independently, at least in triplicate. The data are presented as the mean ± standard deviation. The data were analysed using one-way ANOVA, and Duncan’s test was used to compare overall differences (*P* < 0.05).

## Data Availability

All data included in this study are available on request from the corresponding author.

## List of abbreviations

MTT: 3-(4,5-Dimethylthiazol-2-yl)-2,5-diphenyltetrazolium bromide
ECM: Endothelial cell medium
MOI: Multiplicity of infection
PBS: Phosphate buffered saline
PCR: Polymerase chain reaction
